# Temporal changes of spinal microglia in murine models of neuropathic pain: a scoping review

**DOI:** 10.3389/fimmu.2024.1460072

**Published:** 2024-12-06

**Authors:** Simran Dhir, Hannah Derue, Alfredo Ribeiro-da-Silva

**Affiliations:** ^1^ Department of Pharmacology and Therapeutics, School of Biomedical Sciences, Faculty of Medicine and Health Sciences, McGill University, Montreal, QC, Canada; ^2^ Alan Edwards Centre for Research on Pain, McGill University, Montreal, QC, Canada; ^3^ Integrated Program in Neuroscience, McGill University, Montreal, QC, Canada

**Keywords:** microglia, neuropathic pain (NP), mouse model, spinal cord, temporal changes

## Abstract

Neuropathic pain (NP) is an ineffectively treated, debilitating chronic pain disorder that is associated with maladaptive changes in the central nervous system, particularly in the spinal cord. Murine models of NP looking at the mechanisms underlying these changes suggest an important role of microglia, the resident immune cells of the central nervous system, in various stages of disease progression. However, given the number of different NP models and the resource limitations that come with tracking longitudinal changes in NP animals, many studies fail to truly recapitulate the patterns that exist between pain conditions and temporal microglial changes. This review integrates how NP studies are being carried out in murine models and how microglia changes over time can affect pain behavior in order to inform better study design and highlight knowledge gaps in the field. 258 peer-reviewed, primary source articles looking at spinal microglia in murine models of NP were selected using Covidence. Trends in the type of mice, statistical tests, pain models, interventions, microglial markers and temporal pain behavior and microglia changes were recorded and analyzed. Studies were primarily conducted in inbred, young adult, male mice having peripheral nerve injury which highlights the lack of generalizability in the data currently being collected. Changes in microglia and pain behavior, which were both increased, were tested most commonly up to 2 weeks after pain initiation despite aberrant microglia activity also being recorded at later time points in NP conditions. Studies using treatments that decrease microglia show decreased pain behavior primarily at the 1- and 2-week time point with many studies not recording pain behavior despite the involvement of spinal microglia dysfunction in their development. These results show the need for not only studying spinal microglia dynamics in a variety of NP conditions at longer time points but also for better clinically relevant study design considerations.

## Introduction

Pain, while evolved to serve a protective purpose, can become persistent and lead to maladaptive changes in the central nervous system (CNS). Neuropathic pain (NP) is one such debilitating, chronic pain condition resulting from damage to the somatosensory nervous system, which affects 7%–10% of the population (1). This chronic pain is characterized by symptoms including increased sensitivity to stimuli (hyperalgesia) and a pain response to non-painful stimuli (allodynia) (1). The treatments for NP are largely ineffective and are associated with harmful side effects (1), due to which there is a growing interest in better characterizing this disease and discovering new therapeutic targets.

Microglia, the resident CNS immune cells, are one such target that are garnering popularity and are being studied in murine models of NP due to their involvement in pain progression (2). While inhibition of microglia can suppress mechanical allodynia in NP models (3), globally targeting microglia can be detrimental, particularly due to their role in maintaining CNS homeostasis. This has led to an increase in the study of microglia-mediated pathways in pain conditions. Researchers have found that several dynamic changes can occur in these cells during the different stages of NP development (2). These include the microglia being activated after nerve injury and promoting NP through mechanisms such as the activation of brain-derived neurotrophic factor (BDNF)/TrkB signaling, leading to a disrupted neuron chloride homeostasis and the subsequent decrease in inhibitory transmission (4) or synaptic pruning (5).

This scoping review characterizes the changes in spinal microglia over time in murine models of chronic pain by consolidating the findings on how these microglial changes over time after NP are induced. This study also evaluates the importance of the time points that different studies used to measure chronic pain and the relation of potential pain treatments to spinal microglia and pain development. Overall, this review examines the temporal changes in microglial dynamics and the relationships that exist between pain conditions and microglial activation.

## Materials and methods

A literature search to investigate the changes in the spinal microglia over time in murine models of NP was conducted. A comprehensive review of four minimally overlapping databases (i.e., EMBASE, Web of Science, SCOPUS, and PsycInfo) was completed to ensure that no relevant sources were excluded by this investigation.

### Search strategy

Sources were required to meet the following inclusion criteria: 1) must have been conducted using a mouse model of pain; 2) must have included investigation of microglia in the spinal cord; 3) must be published in the English language in a peer-reviewed journal; and 4) must be a primary source article.

Studies were excluded if they were gray literature sources (i.e., conference proceedings, poster abstracts, etc.). The articles retrieved in the search were also excluded if they were review articles or if the full text was not available.

### Study selection

**Figure 1:**
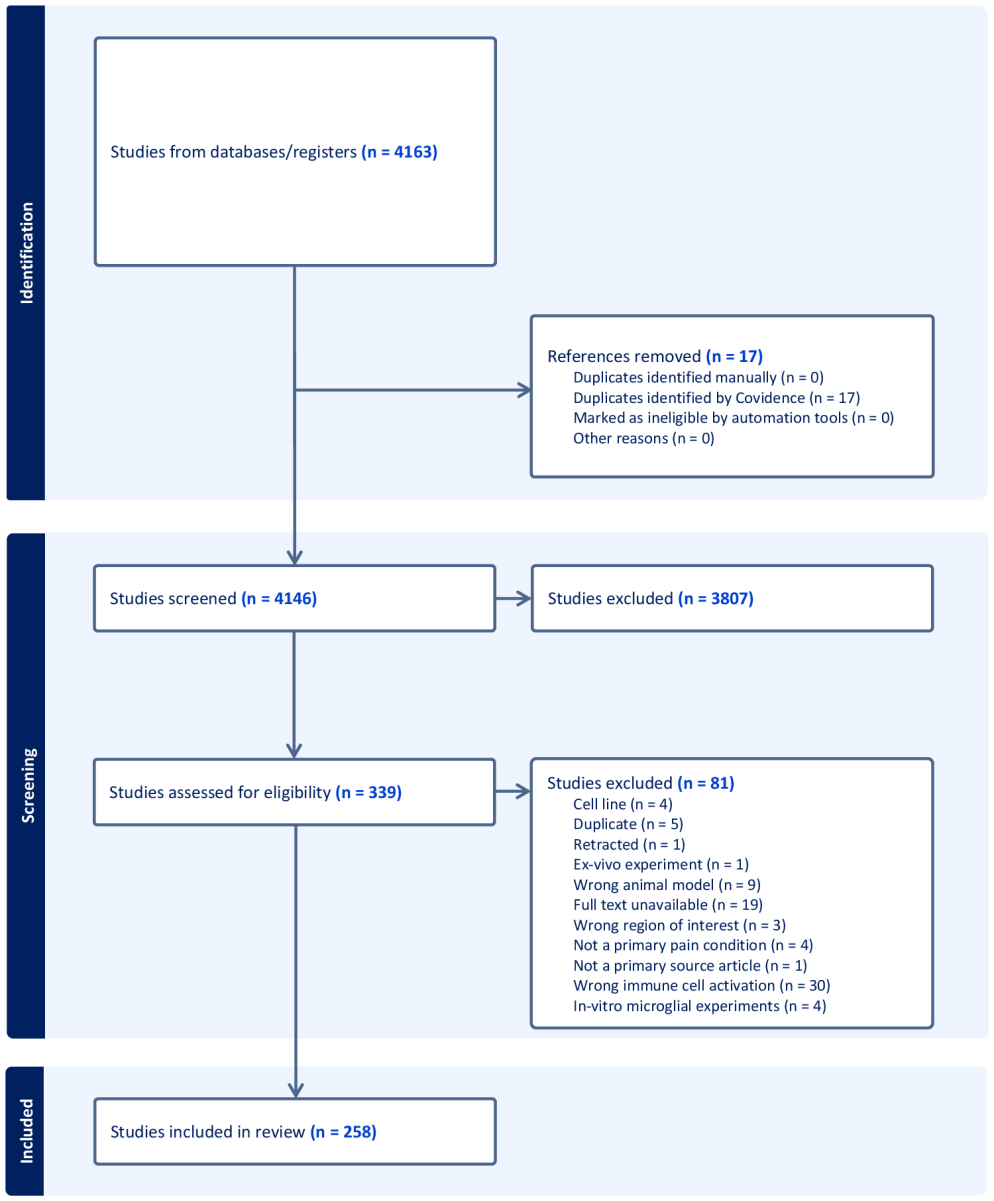
PRISMA flowchart of the study selection, review criteria, and reasons for study exclusion at various phases of the scoping review.

After the initial search of these four databases, 4,146 non-duplicate sources were identified for the screening of titles and abstracts. Following the title and abstract screening of these sources according to the above inclusion and exclusion criteria, 339 sources were identified for a review of the full texts (6–263). Of these sources, only 258 studies were deemed eligible for inclusion in the review based on a dual-reviewer screening process. The reviewers were the first two authors (**Figure 1**). Conflicts at each stage were discussed between reviewers before consensus was reached. Wrong immune cell activation was chosen as a reason for exclusion if the study looked at macrophages, indirect microglia activation markers such as inflammatory cytokines, or only showed colocalization with microglia and not microglial change. Multiple sclerosis (MS) and amyotrophic lateral sclerosis (ALS), disabling chronic CNS diseases affecting spinal cord neurons (264), were included in the study, while studies looking at temporal microglial changes only in rats or cell lines were excluded.

### Data extraction and analysis

Data were extracted from the full-text articles using Covidence’s non-intervention data extracting tool (265). The study characteristics including the publication year, publishing journal, year of publication, sex, sample size, strain, age (distributed into five categories) (266), and the statistical test used were recorded. The type of pain model (267), the treatment, the spinal cord region, the spinal cord section, and the microglial markers were documented. The change in microglia and the pain-related behavior before and after treatment, as well as the time point and the region of the spinal cord where the change occurred, were separated according to the sex of the mice studied. Inter-rater reliability for the full text and title and abstract screening was calculated by Covidence using Cohen’s kappa statistic. The data were exported into Microsoft Excel, where each time point was treated as a separate experiment. The male and female data were separated if both sexes of mice were used in the study and showed different results. The sorting and count functions were then used to separate the data according to pain condition, sex, microglial changes pre- and post-treatment, and mechanical allodynia change post-treatment. All data were presented as counts and percentages. Further information can be found in the **Supplementary Excel File**.

## Results

### Study selection

Moderate inter-rater reliability (268) was found for the full-text and title and abstract screening, with Cohen’s kappa values of 0.53804 and 0.66311, respectively.

### Study characteristics

**Figure 2:**
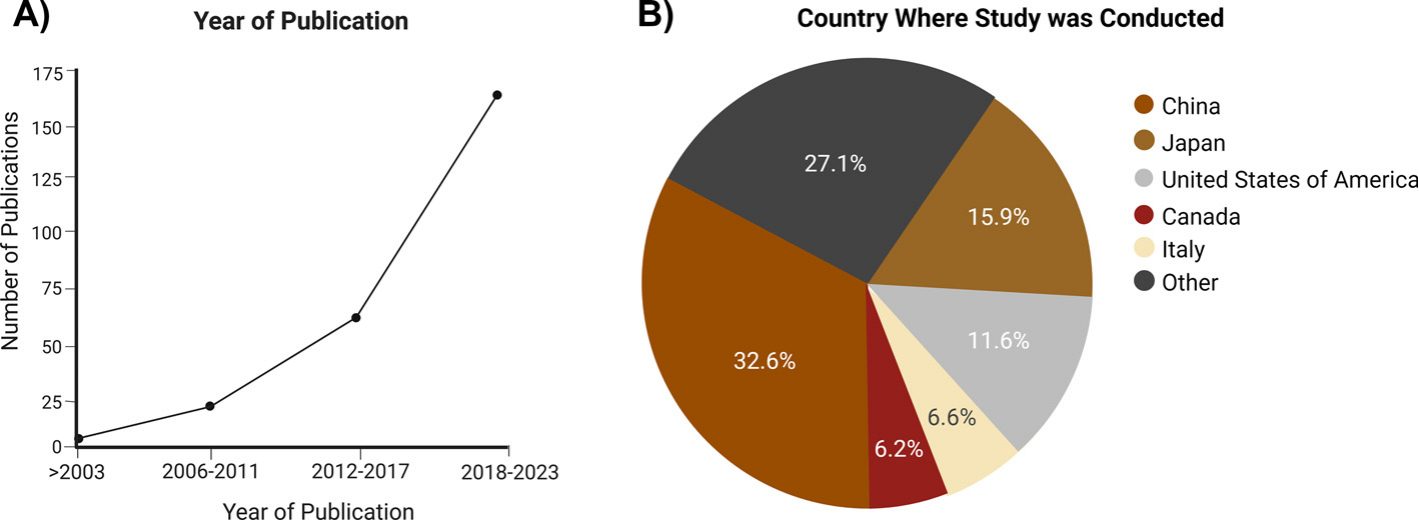
Characteristics of the publications that met the inclusion criteria. **(A)** Year of publication of the included studies. **(B)** Country where the study was conducted.

China was the highest producer of articles (33%), while the second highest, Japan, produced less than half of the percentage of studies conducted in China, which was then followed by the United States of America (**Figure 2B**; **Supplementary Table S1**).

Articles were found in various journals, with Pain, The Journal of Neuroscience, and Molecular Pain being the top 3 publishing sources, but cumulatively only producing 18% of all the included sources.

Despite the literature search within each database not including any limitations on the year of publication, 65.89% of the sources that met the final inclusion criteria were published between 2017 and 2023 (**Figure 2A**).

Only 6% of the studies mentioned testing for normality in their statistical analysis section, while approximately 5% of the studies used non-parametric tests without mentioning a normal distribution test. The average sample size was six mice in each group. Of the studies, 6% had undefined statistics.

### Mouse characteristics

**Figure 3:**
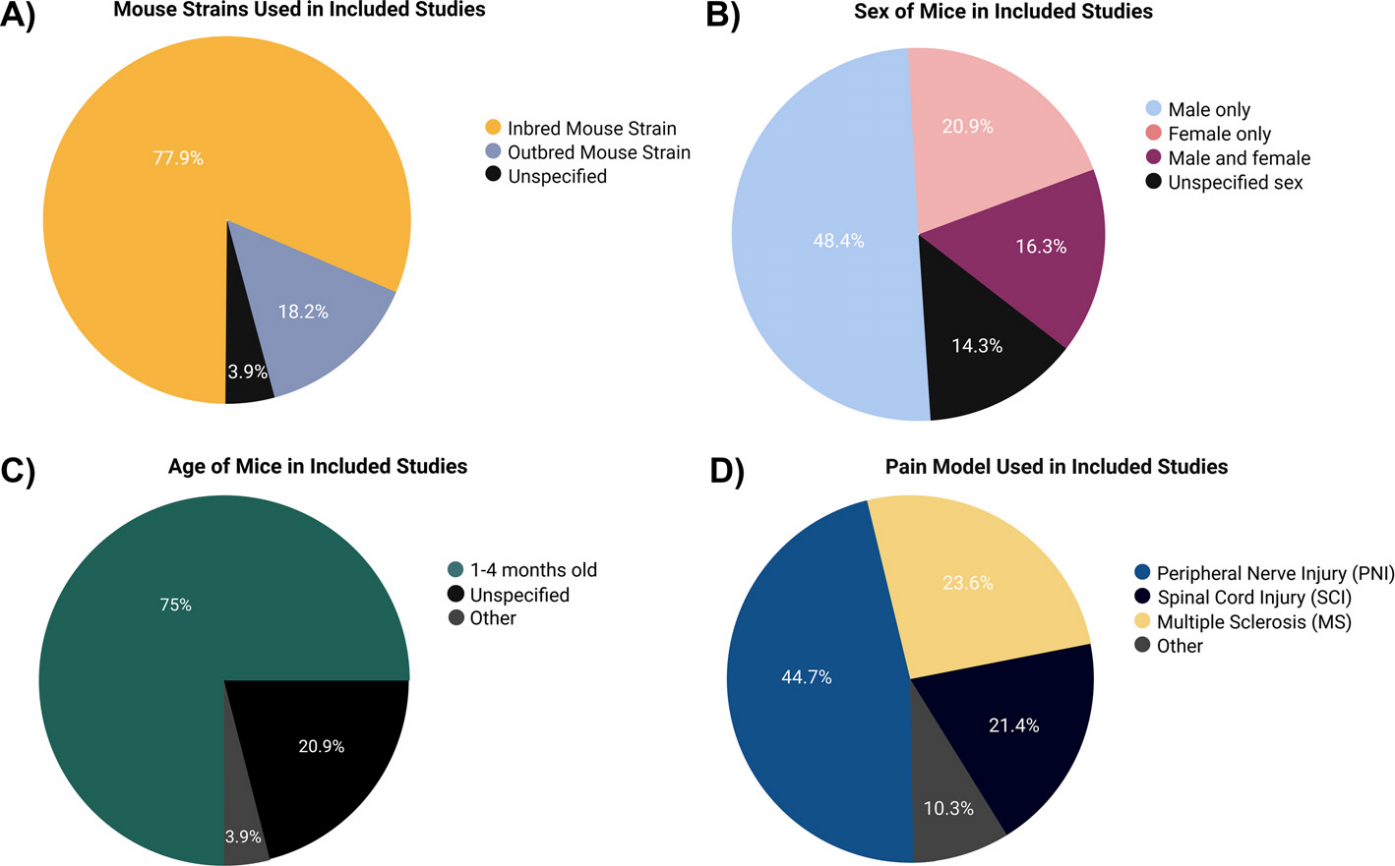
Characteristics of the mice in the studies that met the inclusion criteria. **(A)** Mouse strain. **(B)** Sex. **(C)** Age of mice. **(D)** Pain models applied.

More than twice the number of studies were conducted only in male mice (48%) as compared with female mice (21%), with only 16% studying both sexes. Of the studies, 14% did not specify the sex of the mice used (**Figure 3B**).

Approximately 75% of the studies were carried out using young adult mice aged 1–4 months, while 20% of the studies did not mention the age of the mice used (**Figure 3C**). Of the studied mice, 78% were inbred strains, with 70% of the studies including mice with a C57BL/6 background (**Figure 3A**).

### Models of pain

Peripheral nerve injury (44%), spinal cord injury (SCI; 22%), and MS (9%) were the top 3 pain conditions studied (**Figure 3D**). For a full list of the various models of pain used, see **Supplementary Table S2**.

### Microglial markers

Ionized calcium-binding adapter molecule 1 (Iba-1) was the most prominent microglial marker studied (63%), followed by cluster of differentiation receptor 11b (CD11b; 17%) and fractalkine receptor (CX3CR1; 12%) (**Table 1**). These microglial markers were primarily assessed using immunofluorescence assays.

**Table 1:** Distribution of studies based on the microglial markers used (total of 339 as some studies used more than one microglial marker).

Microglial marker	No. of experiments	Percentage (total = 339)
Iba-1	213	62.8
CD11b	59	17.4
Unspecified	39	11.5
CX3CR1	21	6.2
iNOS	4	1.2
Arg-1	4	1.2
CD11b/c	2	0.6
P2Y12	2	0.6
F4/80	1	0.3
CD68	1	0.3

*Iba-1*, ionized calcium-binding adapter molecule 1; *CD11b*, *CD11b/c*, *CD68*, cluster of differentiation receptor; *CX3CR1*, CX3C motif chemokine receptor 1; F4/80, cell surface glycoprotein; *Arg-1*, arginase 1; *P2Y12*, P2Y purinoceptor 12; *iNOS*, inducible nitric oxide synthase.

### Microglia after pain model induction

**Figure 4:**
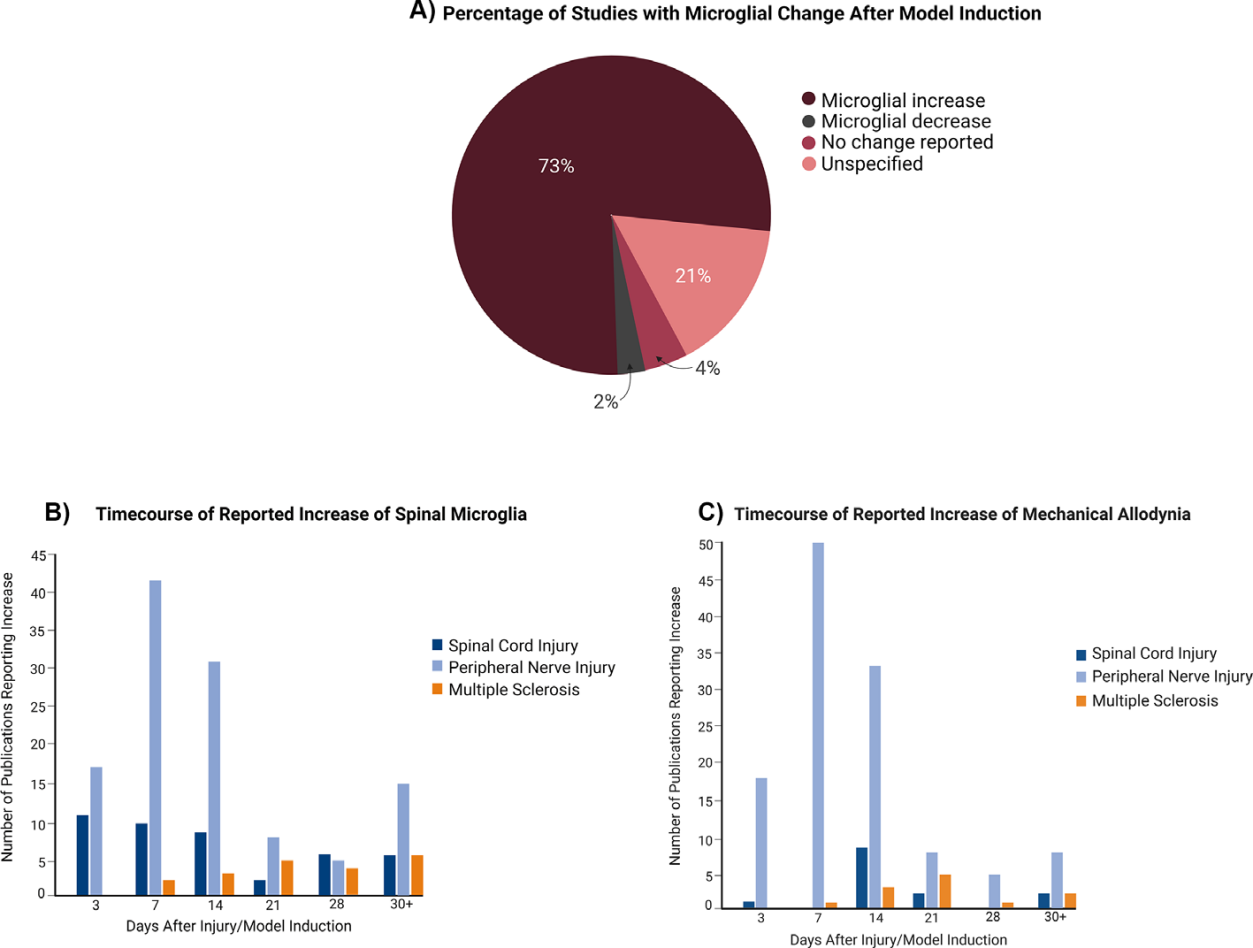
Changes to microglial presence in the spinal cord over time in relation to pain model induction. **(A)** Global (all pain conditions grouped) changes to microglial presence following induction of the pain condition. **(B)** Distribution of studies depending on microglia increase at particular time points. **(C)** Distribution of studies depending on mechanical allodynia increase at time points.

An increase in microglia post-pain initiation was observed in 73% of the experiments, with 21% not specifying any change to microglial presence (**Figure 4A**). The most used time points were days 7, 14, 3, 21, and 28 post-lesion (**Supplementary Table S4**). At every time point, the studies showed an increase in microglia (**Figure 4B**) and mechanical allodynia (**Figure 4C**), with the highest number of studies being peripheral nerve injury experiments conducted within 2 weeks of pain development.

### Microglia post-treatment

**Figure 5:**
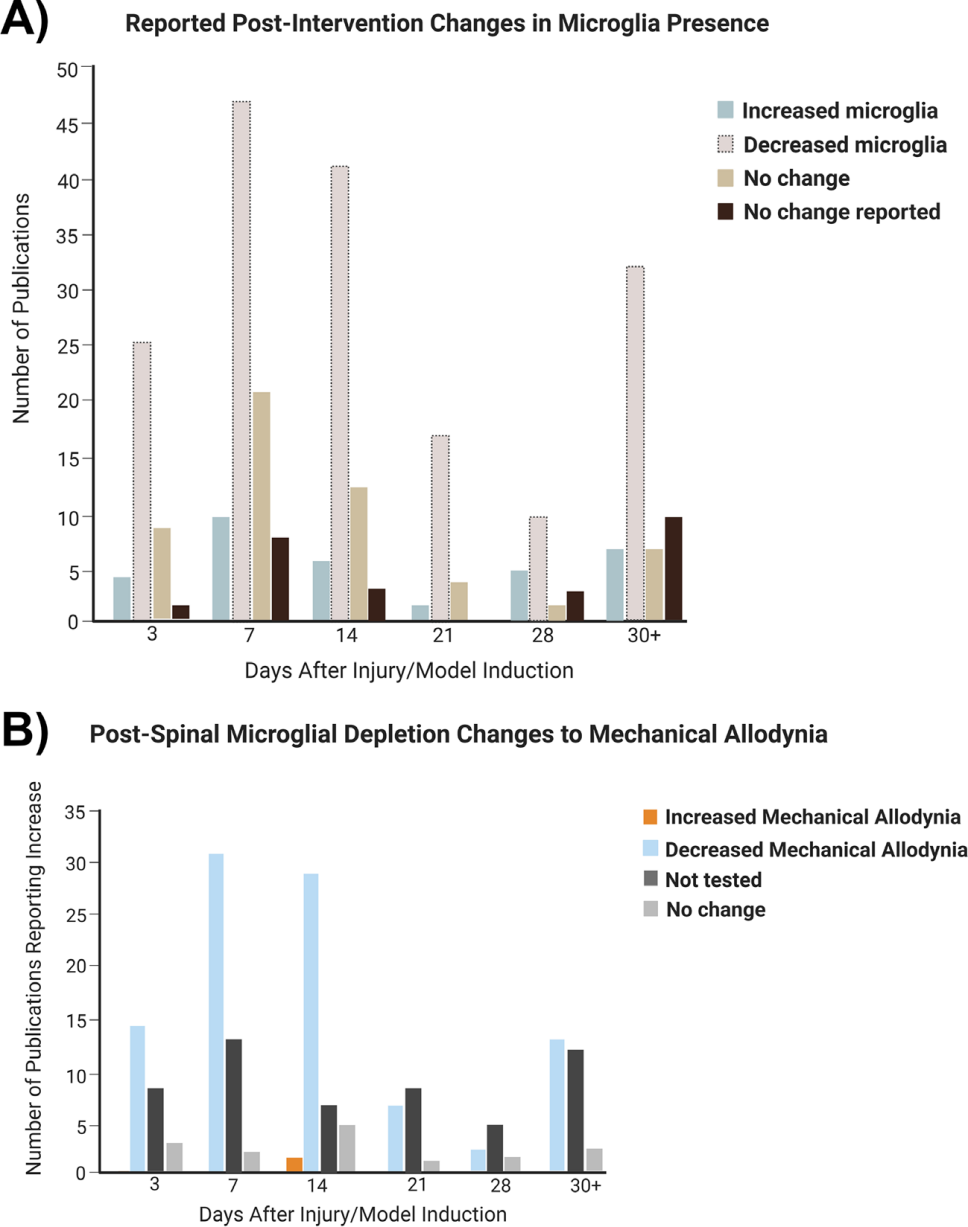
Changes to microglial presence and pain behavior in the spinal cord over time post-treatment (all treatments grouped). **(A)** Distribution of experiments depending on microglial change after treatment. **(B)** Distribution of studies depending on microglial decrease at particular time points.

Post-treatment, spinal microglia decreased at every time point (**Figure 5A**). Up to 2 weeks after injury, mechanical allodynia was primarily decreased post-spinal microglial depletion (**Figure 5B**). After 2 weeks, a similar number of experiments showed decreased mechanical allodynia and unspecified changes to pain behavior despite spinal microglial decrease post-treatment. For a full figure illustrating the various treatments summarized in these figures, see **Supplementary Figure S1**.

## Discussion

The role of spinal microglia in murine NP conditions has recently been the focus of widespread interest from the pain research community given the increasing trend of using mouse models, instead of rats, in pain research (269). This is likely motivated by the advent of using transgenic mouse lines (270) to study the various aspects of chronic pain. An overwhelming majority of the sources that met the eligibility criteria for this review were published within the last 5 years, further reinforcing that this is an area of heightened research interest in the field of preclinical chronic pain research.

Analysis of the characteristics of experimental mice showed that over three quarters of the studies were conducted on inbred mice despite evidence showing that the use of outbred mice is more efficient, generalizable, and clinically relevant (271). Most of the included studies were conducted using only a single mouse strain despite evidence suggesting strain-specific immune responses in NP murine models (92). Young adult mice within 4 months of age, corresponding to approximately 15–20 human years (272), were the most used in these studies, and 2 weeks post-chronic pain initiation was the most studied time point. This can lead to an incomplete understanding of immune cell-mediated pain mechanisms due to mice not being studied for longer time points and at more mature ages (270), especially when microglia have been shown to cause time-dependent changes in NP conditions (5).

Moreover, more than twice the number of studies used only male mice as compared with females, despite chronic pain being more prevalent in female individuals (273). The studies that did use only female mice were skewed toward MS pain models, where female mice better recapitulated the disease phenotype (274). Proliferation of microglia occurred in both sexes following nerve injury (2, 275, 276). Moreover, in this study, we found that, after chronic pain initiation, there is a predominant consensus of an increase in spinal microglia. The role of spinal microglia in chronic pain progression is still contentious, especially with the emerging understanding of the heterogeneity in the microglial phenotypes and their involvement in different pain conditions. Some studies showed that microglia-mediated pain hypersensitivity post-nerve injury is observed primarily in male rodents (275), with other studies finding that microglia are involved in the progression of chronic pain in both sexes (200). This further highlighted the need to include both sexes of mice in chronic pain studies and to properly report the strain, age, and sex of the animals used in the experiments.

The top 3 microglial markers—intracellular protein Iba-1 and the microglial membrane proteins CD11b and CX3CR1—are activated microglial markers that mediate phagocytosis and pathogen recognition, respectively. However, these are general microglial markers that do not differentiate between resident CNS microglia and peripheral macrophages. The use of more specific markers for microglia, such as the transmembrane protein 119 (TMEM119) and purinergic receptor P2Y12R, or co-staining with specific resident macrophage markers, including the high expression of cluster of differentiation receptor (CD44 and CD206), will be required in future studies to properly parse out which immune cells are changing in NP (277). Moreover, microglia can undergo drastic morphological and functional changes when activated by different signals (278). These changes include the M1-like phenotype characterized by the release of inflammatory cytokines and the M2-like phenotype associated with anti-inflammation mediators (278). In addition to NP, many CNS disorders, such as stroke, Alzheimer’s disease, and MS, have shown how specific microglia can be associated with particular stages of disease progression (278). Moreover, transcriptomics data have shown phenotypic profiles of microglia that go beyond the simple M1- and M2-like classification and how some microglia can express multiple markers simultaneously (278). The metrics often applied in the existing literature, including microglial increase/decrease, are crude measures of the nuanced roles that microglia play in the progression of NP. Future studies thus require in-depth analysis of the changes to microglial morphology and function with respect to time and disease progression. Follow-up experiments using specific microglial markers, including the enzymes inducible nitric oxide synthase (iNOS) and arginase-1 (Arg-1), to identify pro- and anti-inflammatory states, respectively, and the accompanying morphological changes are paramount to properly characterize the role of microglia in NP (277, 278).

A majority of the NP studies included in this review were conducted using peripheral nerve injury models, specifically those involving the sciatic nerve. This is likely a result of this model being easily reproducible and having highly localized mechanical allodynia (270). However, there are a plethora of other clinically observed NP conditions (279) caused by drugs, other diseases such as cancer and diabetes, and CNS injury, in which microglial changes and their influence on pain behavior are understudied. In this review, SCI studies frequently examined locomotor dysfunction, while MS models primarily focused on the physical indicators of disease progression, with minimal inclusion of pain behavioral assays, which could provide useful insights into neurodegenerative conditions with a pain component. In the studies in which pain behavior was explicitly quantified, many included measures of evoked mechanical allodynia (primarily via the Von Frey test), choosing to omit clinically relevant measures of other forms of pain, including heat hypersensitivity, cold allodynia, and spontaneous measures of pain (280).

Increased microgliosis and mechanical allodynia was observed in the majority of studies within 1 month of NP initiation, which is consistent with the emerging role of spinal microglia promoting increased pain behavior through the activation, proliferation, and the release of inflammatory immunomodulators post-pain initiation (2). Studies using treatments that decrease spinal microglia were mostly associated with decreased mechanical allodynia up to 2 weeks. After the 2-week time point, the number of studies examining spinal microglial dynamics and studies examining pain behavior declined. This is important to consider because, in conditions such as SCI and peripheral nerve injury, 6 weeks post-injury is considered chronic (281) and microgliosis can occur 20 days after the injury (5), respectively. Moreover, further work must be done to study the long-term potential of microglia-modifying treatments in the reduction of pain behavior.

While microglial involvement in chronic pain has garnered much preclinical focus mechanistically and therapeutically, the manipulation of microglia for chronic pain treatment is not exclusive to animal studies. Human clinical trials using drugs that prevent microglia activation and that reduce inflammation are underway to treat chronic pain diseases such as fibromyalgia (282–286). Animal studies, thus, have the potential to provide a basis for discovering therapeutic targets that can be translated to human research, and the clinical translatability increases with well-designed preclinical studies. In addition, research further elucidating the complexity of microglia-mediated cell signaling during chronic pain continues to provide new methods of targeting microglia, and this can be seen not only through the latest papers being published in the field (287–292) but also through the variety of microglial treatments used in these studies, which included genetic modifications using gene silencing drugs, direct pharmacological microglial ablation, indirect anti-inflammatory drugs, and physical interventions (**Supplementary Figure S1**).

With regard to the evaluation of statistical testing, a very low percentage of studies tested for normality. Only one study explicitly stated that normality was assumed (198). One study went as far as to use a non-parametric test, but, in the same sentence, stated that a normal distribution was assumed (72). This highlighted a lack of understanding of statistical tests and the assumptions associated with them, particularly when dealing with low sample sizes (293) and variable number of animals in each group (294). This can have long-term consequences on the reproducibility and the generalizability of studies, especially when some studies did not even mention which statistical tests were conducted.

The limitations of this review include generalizing the analyses to all chronic pain conditions, particularly when looking at treatment effects. This was due to the low numbers of studies looking at each subclass of chronic pain models (**Supplementary Table S3**) at various time points, further highlighting the opportunity for future research to look at microglial temporal dynamics in specific subtypes of chronic pain models. Moreover, treatments were generalized based on the microglial changes they caused in the spinal cord and not on the drug class due to the widespread heterogeneity in the treatment types (**Supplementary Figure S1**). For the same reasons, the time point of administration and the dose information were not integrated in this review, but are available in the **Supplementary Excel Master Sheet**. While moderate inter-rater reliability scores were obtained, all conflicts were resolved by both reviewers, and the exploratory nature of the scoping review made it such that conditions like MS were included at the conflict resolution stage along with indirect studies of microglial colocalization being excluded from the analysis. Simplifications for compiling the variable data were made, including generalizing mechanical allodynia to pain behavior. In addition, inflammatory microglial increases were included in microglial increase, and one article showing non-inflammatory microglial increase was deemed as microglial decrease due to the lack of distinction of the microglial phenotypes in a majority of papers, as well as the association of inflammatory microglia with disease progression and non-inflammatory microglia with disease resolution (278). Lastly, the literature search for this review was conducted in December 2023, with the analyses being limited to articles published within that time frame.

**Figure 6:**
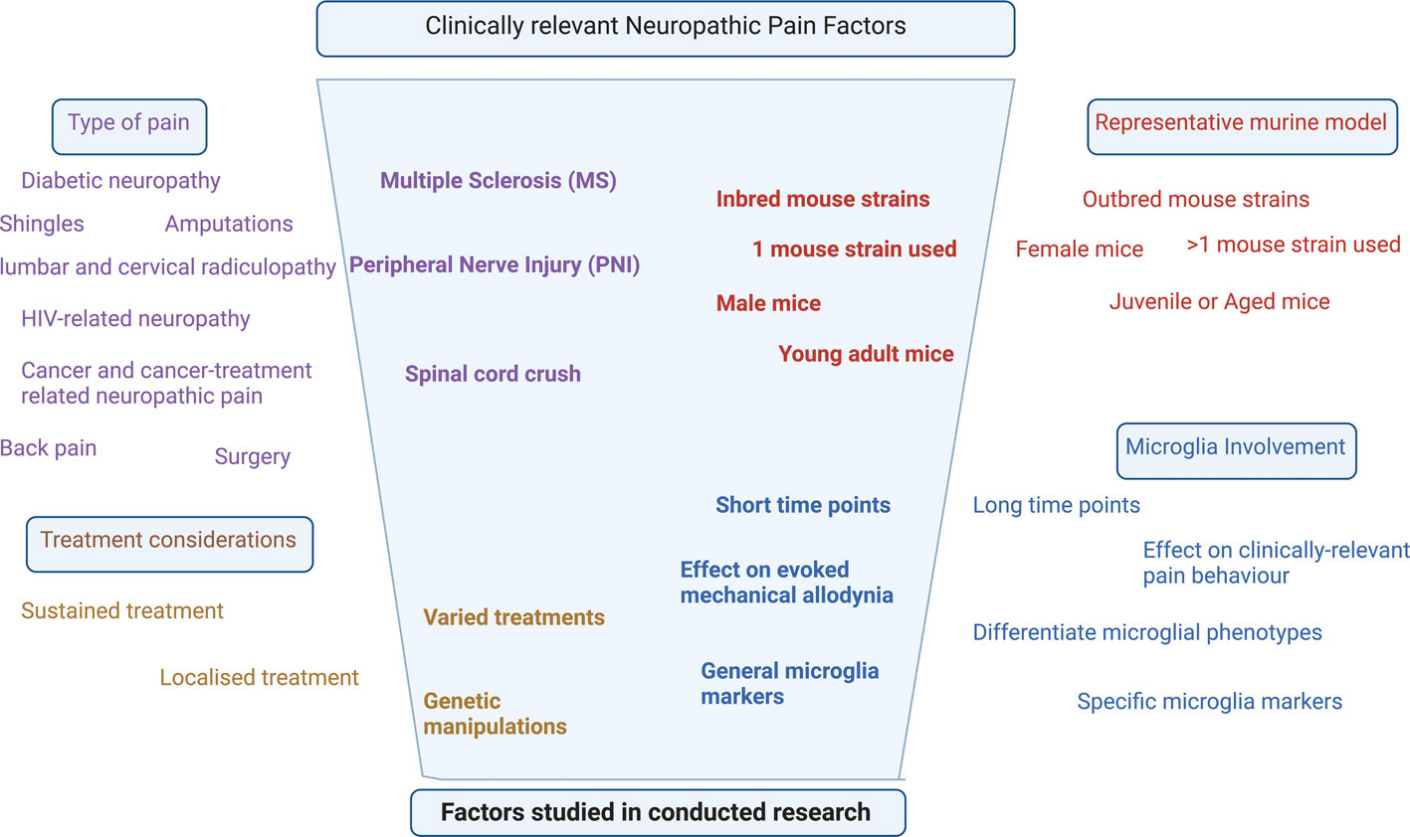
Narrowing down of the factors studied in the majority of research relevant to clinical neuropathic pain.

Temporal changes of spinal microglia are thus becoming increasingly recognized for their key roles in the genesis and maintenance of chronic pain conditions. The importance of microglia is highlighted by their increase at various time points after chronic pain initiation, with most studies in this review showing corresponding mechanical allodynia increases. The decrease in microglia after treatment is related to the decreased mechanical allodynia in some studies; however, many chronic pain studies using SCI and MS pain models did not explicitly study pain behavior, leaving a major gap in pain-relevant studies that could enrich the understanding of the role of microglial dynamics in other chronic pain conditions. This gap in the research, along with the lack of long-term longitudinal studies in specific pain models, highlighted the need for better study design considerations. This includes, but is not limited to, the sex, age, and strain of mice included, the specific microglial markers used, the statistical tests applied, the pain models constructed, the time points assessed, and the pain tests conducted (**Figure 6**).
