# Supplementary material for: Temporal changes of spinal microglia in murine models of neuropathic pain: a scoping review
Source: Front Immunol. 2024 Dec 6;15:1460072. doi: 10.3389/fimmu.2024.1460072 (PMC11671780; doi:10.3389/fimmu.2024.1460072)

Supplemental Tables and Figure:

Supplemental Table S1: Study Characteristics - Summary of characteristics of included studies

| **Study Characteristic** | **Number of Papers** | **Percentage (Total 258)** |
| --- | --- | --- |
| **Year Published** |  |  |
| 2018-2023 | 170 | 65.9 |
| 2012-2017 | 63 | 24.4 |
| 2006-2011 | 23 | 8.9 |
| >2003 | 2 | 0.8 |
| **Country where study was conducted** |  |  |
| China | 84 | 32.6 |
| Japan | 41 | 15.9 |
| United States | 30 | 11.6 |
| Canada | 17 | 6.6 |
| Italy | 16 | 6.2 |
| Republic of Korea | 11 | 4.3 |
| Brazil | 8 | 3.1 |
| Poland | 8 | 3.1 |
| Spain | 7 | 2.7 |
| United Kingdom | 6 | 2.3 |
| Australia | 5 | 1.9 |
| France | 5 | 1.9 |
| Germany | 5 | 1.9 |
| Sweden | 3 | 1.2 |
| Egypt | 2 | 0.8 |
| Kuwait | 2 | 0.8 |
| Austria | 1 | 0.4 |
| Hungary | 1 | 0.4 |
| Israel | 1 | 0.4 |
| Lebanon | 1 | 0.4 |
| Portugal | 1 | 0.4 |
| Republic of China (ROC) | 1 | 0.4 |
| Switzerland | 1 | 0.4 |
| Taiwan | 1 | 0.4 |
| **Age of mice used** |  |  |
| Adolescent (Up to 1 month) | 1 | 0.4 |
| Young Adult (1-4 months) | 194 | 75.2 |
| Mature Adult (4-10 months) | 9 | 3.5 |
| Unspecified | 54 | 20.9 |
|  |  |  |

Supplemental Table S2: Distribution of Pain Conditions Investigated (Total = 349 as some studies looked at more than one pain condition).

| **Pain Conditions investigated** | **Number of Papers** | **Percentage (Total 349)** |
| --- | --- | --- |
| Peripheral Nerve Injury | 152 | 43.6 |
| Spinal Cord Injury | 78 | 22.3 |
| Multiple Sclerosis Model | 32 | 9.2 |
| Inflammatory Pain Model | 18 | 5.2 |
| Diabetes Neuropathic Pain Model | 10 | 2.9 |
| Chemotherapy induced Neuropathic Pain Model | 7 | 2.0 |
| Amyotrophic lateral sclerosis (ALS) Model | 7 | 2.0 |
| Human immunodeficiency virus (HIV)- therapy-induced Neuropathic Pain Model | 5 | 1.4 |
| Cancer Pain Model | 4 | 1.1 |
| Orofacial Pain Model | 3 | 0.9 |
| Cervical compressive myelopathy Mouse Model | 2 | 0.6 |
| Complex regional pain syndrome (CRPS) Model | 2 | 0.6 |
| Familial Amyotrophic lateral sclerosis (ALS) Model | 2 | 0.6 |
| Ischemic spinal cord injury | 2 | 0.6 |
| Limb Fracture | 2 | 0.6 |
| Trigeminal Neuropathic Pain | 2 | 0.6 |
| Unspecified | 2 | 0.6 |
| Burn Injury | 2 | 0.6 |
| Chronic morphine tolerance | 2 | 0.6 |
| Aging | 1 | 0.3 |
| Alcohol induced Pain Model | 1 | 0.3 |
| Cervical sympathetic trunk (CST) | 1 | 0.3 |
| Chronic Hypoxia Model | 1 | 0.3 |
| Electrical stimulation-induced chronic pain model | 1 | 0.3 |
| Fibromyalgia Animal Model | 1 | 0.3 |
| ADAM17 Deficiency | 1 | 0.3 |
| Incisional Pain Model | 1 | 0.3 |
| Lumbar Disc Herniation | 1 | 0.3 |
| PSNL Mimic Model | 1 | 0.3 |
| Psychosocial Stress Model | 1 | 0.3 |
| PTEN conditional knockout mice | 1 | 0.3 |

Supplemental Table S3: Peripheral Nerve Injury Subtypes and their Frequency

| **Type of Peripheral Nerve Injury** | **Number of studies** | **Percentage (Total = 152)** |
| --- | --- | --- |
| CCI | 40 | 26.3 |
| SNI | 37 | 24.3 |
| Spinal nerve transection | 33 | 21.7 |
| partial sciatic nerve ligation (pSNL) | 30 | 19.7 |
| Peripheral nerve injury | 7 | 4.6 |
| Spinal Nerve Ligation | 2 | 1.3 |
| Crush injury | 1 | 0.7 |
| Nucleus pulposus (NP) application | 1 | 0.7 |
| Sciatic nerve cut and repair surgeries | 1 | 0.7 |

Supplemental Table S4: Time points post pain initiation examined for microglia increase and their frequencies (Total = 254 as each time point treated as separate experiment)

| **Day post injury** | **Number of studies with microglia increase** | **Percentage (Total 254)** |
| --- | --- | --- |
| 7 | 68 | 26.8 |
| 14 | 53 | 20.9 |
| 3 | 32 | 12.6 |
| 21 | 16 | 6.3 |
| 28 | 15 | 5.9 |
| 8 | 7 | 2.8 |
| 5 | 6 | 2.4 |
| 10 | 4 | 1.6 |
| 9 | 3 | 1.2 |
| 12 | 3 | 1.2 |
| 15 | 3 | 1.2 |
| 24 | 3 | 1.2 |
| 30 | 3 | 1.2 |
| 42 | 3 | 1.2 |
| not clarified | 3 | 1.2 |
| 18 | 2 | 0.8 |
| 35 | 2 | 0.8 |
| 49 | 2 | 0.8 |
| 54 | 2 | 0.8 |
| 70 | 2 | 0.8 |
| 84 | 2 | 0.8 |
| 4 | 1 | 0.4 |
| 6 | 1 | 0.4 |
| 11 | 1 | 0.4 |
| 13 | 1 | 0.4 |
| 16 | 1 | 0.4 |
| 32 | 1 | 0.4 |
| 37 | 1 | 0.4 |
| 40 | 1 | 0.4 |
| 50 | 1 | 0.4 |
| 98 | 1 | 0.4 |
| 112 | 1 | 0.4 |
| 120 | 1 | 0.4 |
| 121 | 1 | 0.4 |
| 133 | 1 | 0.4 |
| 150 | 1 | 0.4 |
| 154 | 1 | 0.4 |
| 168 | 1 | 0.4 |
| 196 | 1 | 0.4 |
| 517 | 1 | 0.4 |
| 0 | 1 | 0.4 |

**Supplemental Figures**

Supplemental Figure S1: Intervention Methods Applied in Included Studies. A visual representation of the included intervention methods for all studies which met inclusion criteria.


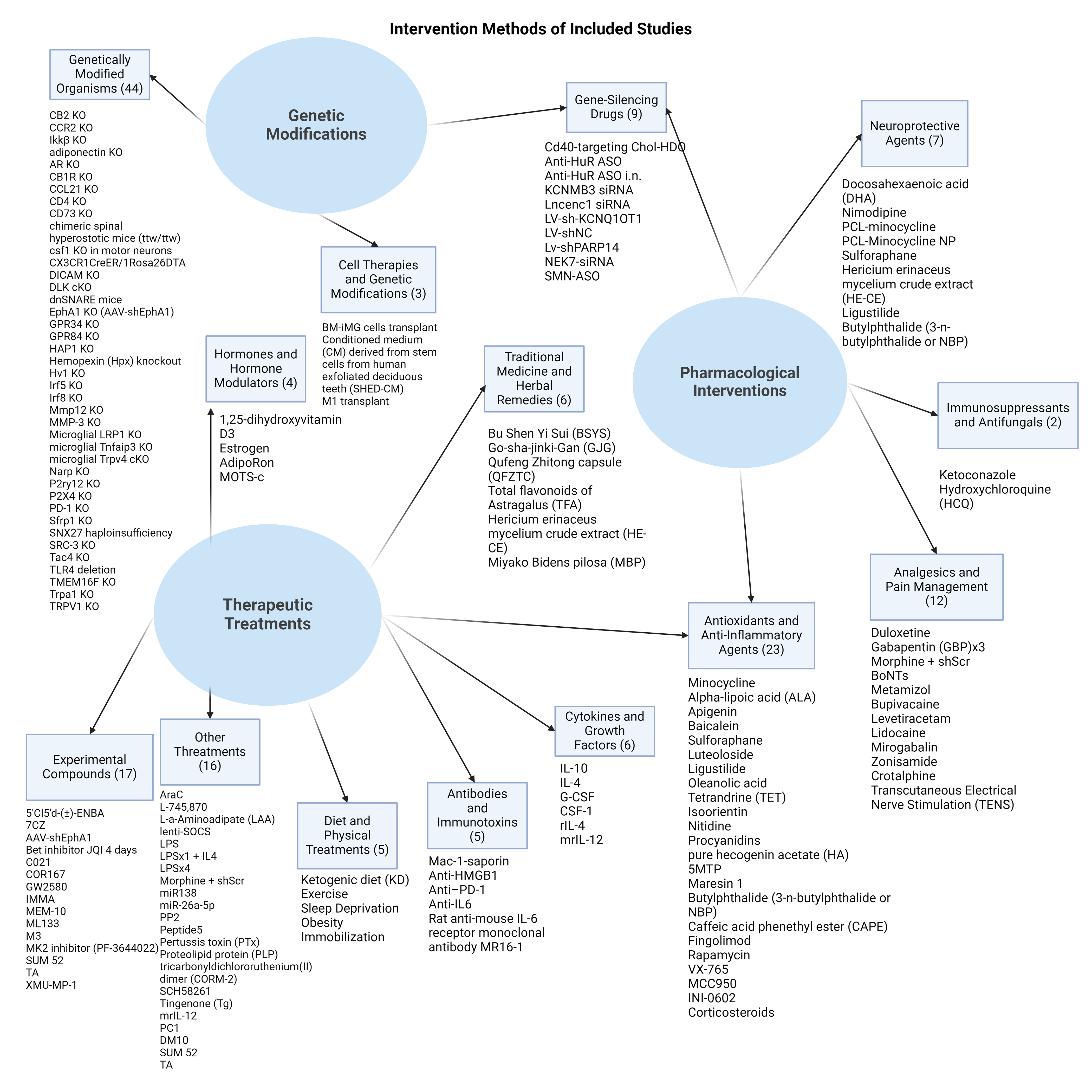

Supplement: Supplementary file 1 [file SupplementaryFile1.docx]
