# Supplementary figures and images for: Temporal changes of spinal microglia in murine models of neuropathic pain: a scoping review
Source: Front Immunol. 2024 Dec 6;15:1460072. doi: 10.3389/fimmu.2024.1460072 (PMC11671780; doi:10.3389/fimmu.2024.1460072)

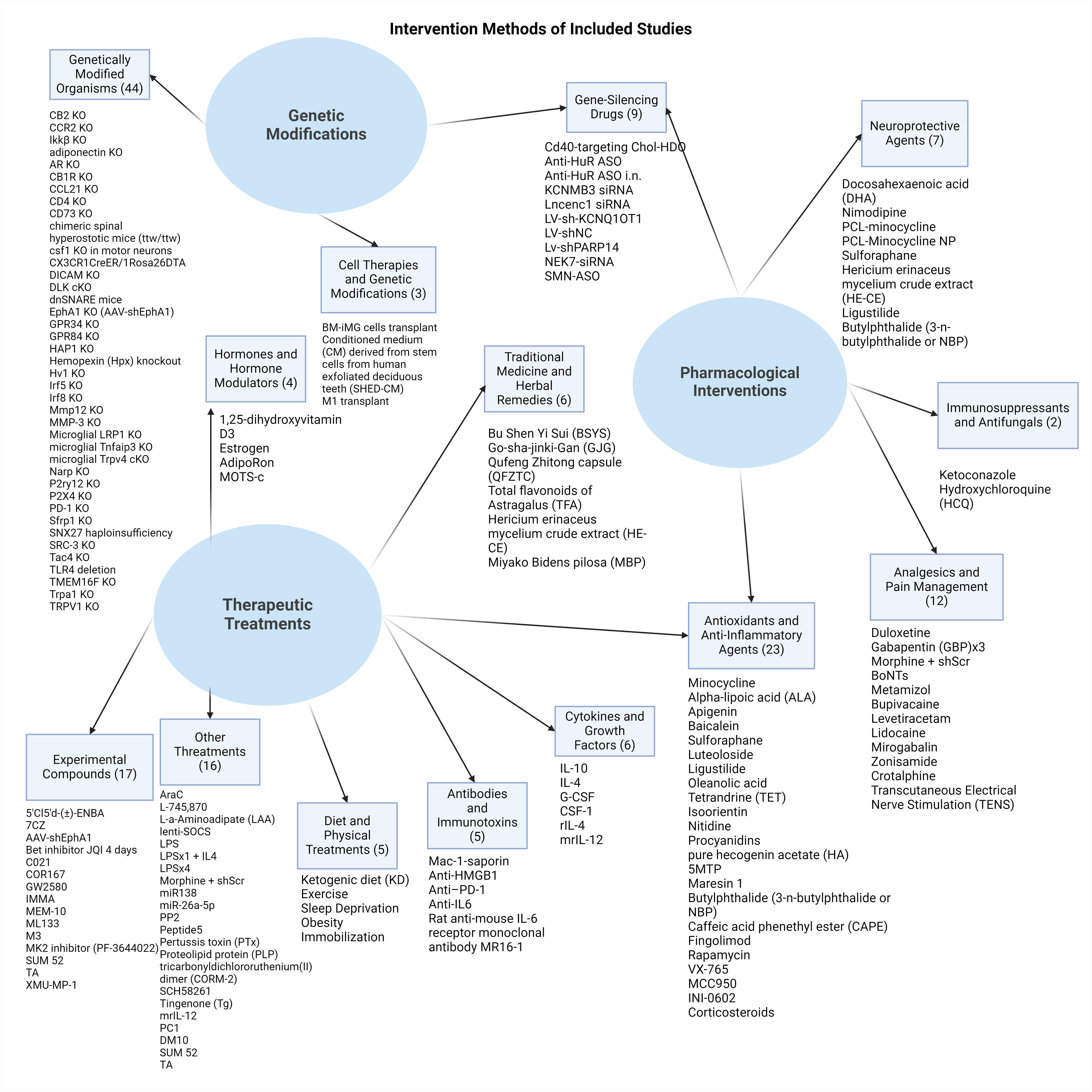

Supplement: Supplementary file 3 [file SupplementaryFigure1.tif]
